# Neutrophil depletion in the early inflammatory phase delayed cutaneous wound healing in older rats: improvements due to the use of un-denatured camel whey protein

**DOI:** 10.1186/1746-1596-9-46

**Published:** 2014-03-04

**Authors:** Hossam Ebaid

**Affiliations:** 1 Department of Zoology, College of Science, King Saud University, Riyadh, Kingdom of Saudi Arabia; 2 Department of Zoology, Faculty of Science, El-Minia University, Minya, Egypt

**Keywords:** Neutrophil recruitment, Inflammatory phase, Wound healing, Whey protein, Aged rat model

## Abstract

**Background:**

While it is known that advanced age alters the recruitment of neutrophils during wound healing, thereby delaying the wound healing process, little is known about prolonged wound healing in advanced ages. Thus, we investigated the correlation of neutrophil recruitment with healing events, and the impact of whey protein (WP) on neutrophil activation.

**Methods:**

The animals were allocated into wounded young group, wounded older group and wounded older rats with daily treatment of WP at a dose of 100 mg/kg of body weight.

**Results:**

Our results pointed to a marked deficiency in the number of neutrophils in the wounds of older rats, which was accompanied with impairment of the healing process. In the group of older rats, phagocytic activity, as tested by fluorescence microscopy, declined throughout the first 24 hours after wounding. Both the neutrophil number and the phagocytic activity recovered in older rats which received WP supplementation. Interestingly, WP was found to significantly up-regulate the MIP-1α and CINC-1 mRNA expression in old rats. On the other hand, the wound size in older rats was significantly higher than that in younger ones. Blood angiogenesis was also significantly delayed in the older group as opposed to the young rats. WP, however, was found to return these indices to normal levels in the older rats. Proliferation and epidermal migration of the keratinocytes and the collagen deposition were also returned to the normal rates.

**Conclusions:**

This data confirms the critical role of neutrophil recruitment in the early inflammatory phase of wound healing in older rats. In addition, WP protein was used to improve neutrophil function in older rats, healing events returned to a more normal profile.

**Virtual slides:**

The virtual slide(s) for this article can be found here: http://www.diagnosticpathology.diagnomx.eu/vs/2100966986117779.

## Background

In the inflammatory phase of wound healing, neutrophils are responsible for microbial clearance in the region of the wound region, playing a role in antigen presentation, phagocytosis and the production of inflammatory cytokines and growth factors [1,2]. The appropriate recruitment and function of these cells are crucial for the efficient removal of microbial agents and, thereby, a normal healing process [3,4].

Wound healing capacity is known to decline with increasing age [5,6] and it has been suggested that age-mediated defects in early neutrophil recruitment may alter the dynamics of the inflammatory phase of wound healing [7]. Stress is also thought to modulate neutrophil and macrophage recruitment, chemokine gene expression and the adhesion molecule expression [8-10]. In young subjects, small numbers of neutrophils may be sufficient for wound recovery, or other cells may have the capability to substitute for neutrophils. In the elderly, however, the effectiveness of neutrophils decreases and other cells are less able to replace them, and therefore greater numbers of neutrophils may be necessary [11].

A further factor that limits wound-healing response rates after injury is the fact that overall signalling efficiency is reduced as a function of age [12]. The persistently high levels of pro-inflammatory cytokines in the wounds of the elderly induce high levels of matrix metalloproteinases (MMPs). This suppresses growth factors, receptors and matrix proteins essential for healing [13,14]. MMPs control the fragmentation of the basal membrane, the induction of inflammation, angiogenesis and re-epithelialization. Ungvari et al., [15] found that age-related impairment of angiogenesis is likely to play a central role in the development of vascular cognitive impairment. Moreover, chemoattractants, which direct the transit of leukocytes out of the mainstream of the blood and into tissues at sites of inflammation, are also reduced. This is significant since neutrophils are uniquely sensitive to a vast array of chemoattractants including MIP-1a, MIP-1b, MIP-2, KC, and others [16].

Although little is known about how to enhance the wound healing process in the elderly we previously confirmed a promising role for the antioxidant whey protein (WP) in the wound healing process using experimental diabetic models [17,18]. The antioxidant activity of WP is most likely linked to its contribution to glutathione (GSH) synthesis. WP provides cysteine, which contains an antioxidant thiol group, combines with glycine and glutamate to form GSH. Here, we investigated whether un-denatured camel WP can accelerate the healing of full-thickness wounds in aged rats by a mechanism that involves improving the neutrophil recruitment. We studied the effect of WP on neutrophil recruitment during wound healing in rats of advanced age.

## Materials and methods

### Preparation of whey proteins

The milk was skimmed by centrifugation at 5000 g for 20 min using an IEC Model K centrifuge [Boston, USA]. Skim milk was acidified to pH 4.3 using 1 M of HCl. The precipitated casein was removed by centrifugation, and the supernatant containing the whey protein was saturated with ammonium sulfate (70% saturation) and incubated overnight at 4°C. The precipitated whey protein was collected by centrifugation and dialyzed against distilled water for 48 h at 4°C using a Spectra/Pro® Membrane, MWCO 6000–8000 Da. The obtained dialyzate was lyophilized using a Unitop 600 SL, [Virtis Company, Gardiner, New York 12525 USA] and were kept at -20°C until use. The dialyzate containing non-denatured whey protein was freeze-dried and refrigerated until use.

### Ethical approval

Camel milk was obtained from a camel breed (Majaheem) from the Najd region (Alazeria farm; GPS: 300 02 47/ 300 02 27) in Saudi Arabia. Specific permissions were not required for activities in this private farm. This study did not involve endangered or protected species. Regarding experimental animals, all procedures were conducted in accordance with the standards set forth in the guidelines for the care and use of experimental animals by the Committee for the Purpose of Control and Supervision of Experiments on Animals and the National Institutes of Health. The study protocol (care and handling of experimental animals) was approved by the Animal Ethics Committee of the Zoology Department in the College of Science at King Saud University.

### Experimental design

Young male albino rats (*Rattus rattus*) (about 6 month old) were purchased from College of pharmacy, King Saud University. Male albino rats (*Rattus rattus*) classified as old rats by College of pharmacy, King Saud University (more than 23 month old) were used in this experiment. The supplemented volume for all groups was constant and did not exceed 1000 μl per dosage per day. The optimal dose of WP was determined in our laboratory on the basis of several established studies and parameters. The animals were allocated into 3 groups of 15 animals each, assigned as follows: Wounded young group (AD) with daily administration of the vehicle (1000 μl/rat/day), PBS by gastric intubation for 2 days (n = 5), 4 days (n = 5) or 8 days (n = 5). Wounded older group (SN) with daily administration of the vehicle (1000 μl/rat/day), PBS by gastric intubation for 2 days (n = 5), 4 days (n = 5) or 8 days (n = 5). Wounded older rats with daily treatment of WP (SNWP) at a dose of 100 mg/kg of body weight (1000 μl/rat/day) by gastric intubation for 2 days (n = 5), 4 days (n = 5) or 8 days (n = 5). Additional supplementary groups such as young rats treated with WP and the non-injuried old rat groups were studied for confirming the results of the three main groups. However, data from these groups are not included in this study.

### Excisional wound preparation

Rats were anesthetized, and the back of the rat was shaved and sterilized using an alcohol swab. The wound biopsy model used in this experiment was performed as previously described [19] with slight modification. The shaved skin was pinched and folded, and the wound was punched through the full thickness of the folded skin to form a 5 mm diameter circle below the shoulder blades of each rat.

### Fluorescence microscopy

To detect the phagocytic activity in the inflammatory phase (4, 8, 24 hours after wounding) of the wounded tissues, fluorescence nano-particles were injected just one hour before sacrificing of rats. Skin pieces were embedded in OCT compound (Sakura, Zouterwede, The Netherlands) and snap frozen on dry ice before serial sections were mounted on superfrost plus slides (Menzel-Glaser, Braunschweig, Germany). Evaluation of fluorescence intensity was performed as previously described [20] using the ImageJ software.

### Morphometric indecies

Wounds from individual rats were digitally photographed every day. A standard circle equivalent in size to the initial wound area was drawn beside the wound and used as a reference. Wound size was calculated by determining the area of the wound each day in comparison to the area of the standard circle. Wound closure was expressed as the ratio of the initial wound size to the wound area (each day after wounding). A higher ratio indicates faster wound closure. Wound width is calculated by the distance between the two opposite leading epidermal ends. The percentage of epidermal cell migration is calculated by the summussion of the long of the two leading ends of the migrated epidermal cells divided on the long of the initial wound distance.

Dermal thickness was measured in five animals from each group and normal skin as the control. A representative image of H&E-stained sections was obtained. The dermal thickness was measured at three points in each image [21]. The blood vessel density of each wound was calculated for each wound (n = 5). Five non-consecutive tissue sections were mad and three randomly selected fields (400× magnification) of wounded tissues were photographed by microscopy (Leica, Wetzlar, Germany). A total of 10 images were taken for each wound, and new vessels were assessed by measuring dermal capillaries in the wound region.

### Blood and tissue sampling

At days 2, 4 and 8 of the after wounding, animals were sacrificed, and blood samples were obtained from the carotid artery. Then, animals were decapitated and dissected, and livers were rapidly excised. Blood samples were left to coagulate and then were centrifuged, and clear non-hemolyzed serum was kept at -20°C until used. Livers were kept at -20°C pending homogenization.

### Estimation of glutathione

Glutathione (GSH) assay was carried out on tissue as previously described [22]. The fresh liver weights were recorded, and organs were frozen until use. A 10% (w/v) homogenate of each frozen tissue was prepared, and the supernatant was used in the enzymatic assays. Glutathione concentrations were measured by adding 100 μl of supernatant to 400 μl PBS [containing 200 mM MCB and 2 U/ml glutathione S-transferase (per 100 μl)]. Glutathione concentrations were then determined by measuring the absorbance of the reaction after 1 min at 340 nm using an UV Visible Spectrometer (Ultrospec 2000, Pharmacia Biotech). Glutathione standards were measured concurrently to obtain a standard curve that was used to calculate GSH concentrations in samples. Results were expressed as μg GSH/g tissue. Statistical comparisons of GSH activities between controls and treatments in each case were performed using Minitab statistical program as detailed below.

### Estimation of lipid peroxidation

The peroxidation of the endogenous lipids in the liver homogenates was estimated spectrophotometrically following the method described by Okhawa et al., [23] and expressed in nanomoles of malondialdehyde (MDA) per milliliter of homogenate (nmole/ml). A 0.5 ml aliquot of the resulting supernatant was shaken with 2.5 ml of 20% trichloroacetic acid (TCA). To the resulting mixture, 1 ml of 0.67% thiobarbituric acid (TBA) was added, and the samples were shaken and incubated for 30 min in a boiling water bath followed by immediate rapid cooling in ice for 5 min. After cooling, 4 ml of n-butyl-alcohol was added, and the samples were shaken well. The resulting mixture was then centrifuged at 16,000 g for 5 min. The resultant n-butyl-alcohol layer was moved into a separate tube, and MDA content was determined spectrophotometrically at 535 nm using an UV Visible Spectrometer (Ultrospec 2000, Pharmacia Biotech).

### ELISA estimation of TNF-α

The level of TNF-α in the serum of experimental groups was determined using specific ELISA kits purchased from R and A Systems, USA. The concentration of TNF-α was determined using a spectrophotometer at 450 nm according to the manufacturers’ instructions.

### Histological analyses

The rats were euthanized with an overdose of isoflurane, and tissue samples were collected from the wound sites to examine neutrophil infiltration into the wound area. Mallory Trichrome staining were applied for detecting the collagen and the iron depositions, in the dermal tissue. The degree of tissue damage was examined blindly using a Leica DMRB/E light microscope (Heerbrugg, Switzerland). Photographs of the sections were taken, and the images were digitized using Adobe Photoshop (Adobe Systems, Mountain View, CA). Wounds were removed from four rats from each treatment group at 6 h, 24 h and at the end of the study period after wounding by cutting a square area that encompassed the entire wound site. The harvested tissues were immediately stored in a 10% formaldehyde solution in phosphate-buttered saline, washed in PBS, dehydrated in series of alcohol dilutions and embedded in paraffin. Microtome sections were cut vertically across the wound site and adhered to slides prior to staining with hematoxylin and eosin. Photographs of the sections in wound site were taken, and the images were digitized using Adobe Photoshop (Adobe Systems, Mountain View, CA). The neutrophil number was determined at 20-random locations within the epidermal and dermal tissues in the wound region for each animal from each group using a Leica Qwin 500 image analyzer.

### Immunochemical detection of proliferated cell nuclear antigen (PCNA)

PCNA staining was performed in 4 μm thick sections and was employed in 0.01 M citrate buffer pH 6. Blocking of non-specific reactivities was performed with 1% normal goat serum and 3% non-fat milk. PCNA monoclonal antibody (Novo Castra NCL-PCNA) was applied in a concentration of 1:100 PBS and then incubated in secondary antibodies. After washing sections were incubated in avidine biotine complex reagents (ABC-kit-Vector) and in peroxidase reagent (3,3’-diaminobenzidine tetrahydrochloride, Sigma) containing 0.01% H_2_O_2_ in PBS [24]. Photographs of the sections were taken, and the images were digitized using Adobe Photoshop (Adobe Systems, Mountain View, CA). The PCNA-stained cells (the number of red brown stained cells) was determined at 20-random locations within the epidermal and dermal tissues in the wound region for each animal from each group using a Leica Qwin 500 image analyzer.

### RNA extraction and reverse transcriptase PCR (RT-PCR)

RNA was extracted from the collected samples using RNeasy Mini Kit (QIAGEN), according to the manufacturer instructions. RNA extracts were used as templates to detect the expression of several genes. RT-PCR was performed using QIAGEN OneStep RT-PCR kit as instructed in the manufacturer’s instruction manual.

### Statistics

The statistical analysis was performed using the MINITAB software (MINITAB, State College, PA, Version 13.1, 2002). The data were normally distributed with homogeneous variances. Thus, the one-way ANOVA statistical measure was used to determine the overall effect of each treatment. This measure was supplemented by individual comparison between the different treatments using Tukey’s method for pairwise comparisons. The results were expressed as mean (M) ± standard deviation (SD). Only statistically significant differences with P < 0.05 were found between the treatment group and the control, and between the treatment group and the aged group considered.

## Results

### Effect of WP on the wound closure, morphometeric indices and oxidative status

**Figure 1:**
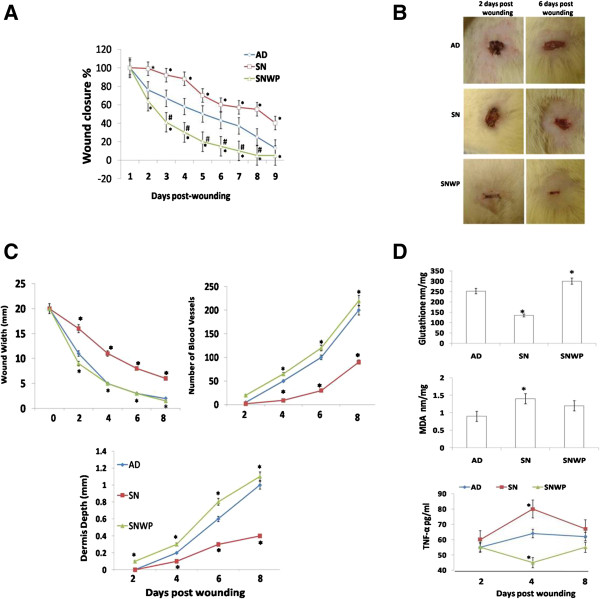
**Wound closure rate, morphometric indices and oxidative stress. A**: Percentage of wound closure rate in control (AD), aged (SN) and aged rats supplemented with WP (SNWP). **B**: Representative external photograph showing wounds in the different groups at the second and sixth days after wounding. Wound contraction is obvious in the SNWP animals at the second day after wounding. **C**: Morphometric indices of the wounds from three different groups for eight days following wounding. **D**: Glutathione and MDA levels in different groups two days after wounding. Results of the four and eight days were approximately the same of two days (data not shown). Lower is the TNF-α levels in different groups. Values shown are means ± SD. *shows the significance in comparison with the control group. # shows the significance in comparison with the older group.

External changes in the wound morphology were monitored daily during the experimental period. The percentage of older rats exhibiting wound closure was significantly lower than that of the control rats. WP was found to recover the wound closure rate in the older rats to a similar level to that of the young rats (Figure 1A,B).

To monitor the progress of wound healing, morphometeric indices were evaluated at two day intervals. The size of the wound opening was significantly higher in sections from older rats in comparison to those from the young rats. The wound opening of older rats supplemented with WP was gradually minimized, however, to become completely closed by the eighth day after wounding. In addition, the number of new blood vessels and the depth of the dermal tissue in the wounded region were significantly reduced in older rats in comparison to the younger ones, but WP was found to significantly activate angiogenesis and the construction of dermal constituents (Figure 1C).

In the inflammatory phase, older rats showed a significant decrease in GSH level compared to the control group, while those older rats supplemented with WP displayed significant improvements in this level. Older rats showed a very sharp increase in the level of MDA compared to the young rats. Older rats supplemented with WP, however, showed a significant decrease in the level of MDA compared to their untreated older counterparts (Figure 1D).

Inflammatory cytokines play a crucial role in wound healing and thus, we estimated TNF-α concentration in different rat groups. Older rats showed a significant increase in TNF-α level compared to the control group, while those older rats supplemented with WP displayed significant decrease in this level at the 4^th^ day after wounding (Figure 1D).

### Effect of WP on neutrophil chemoattractants, infiltration and phagocytic activity

**Figure 2:**
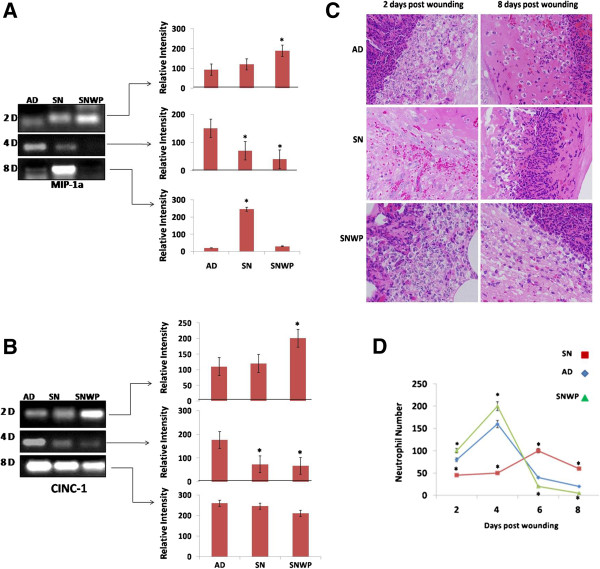
**Neutrophil chemoattractants and wound infiltration.** MIP-1α **(A)** and CINC-1 **(B)** mRNA expression in wounded skin from the different groups. **C**: Representative skin sections showing neutrophil infiltration during the inflammatory and remodelling phases of wound healing (H&E, × 400). **D**: Changes in the number of neutrophils during the experimental period (between the second and eighth days after wounding) for the different groups. Values shown are means ± SD. *shows the significance in comparison with the control group.

To detect the neutrophil chemoattractants in the wounded cutaneous tissues, we estimated the MIP-1α and CINC-1 mRNA (Figure 2A,B). Results showed that WP was found to significantly up-regulate the MIP-1α (Figure 2A) and CINC-1 (Figure 2B) mRNA expression in old rats supplemented with WP at 2 days after wounding with respect to old rats, in which MIP-1α and CINC-1 mRNA expression was delayed to the 8^th^ day after wounding.

To evaluate neutrophil infiltration rate and timing, we investigated the extent of this infiltration on the second, fourth and eighth days after wounding (Figure 2C,D). Neutrophils, were clearly observed in great numbers in the dermis of the young rats at the second day after wounding (Figure 2C,D), indicating the critical role of these cells in this phase, but their numbers were found to gradually decrease towards the end of wound healing (by the eighth day after wounding). In contrast, in the older rats, a lower number of neutrophils infiltrated the dermis in the period up till two days after wounding but this number then gradually increased to reach a peak on the eighth day after wounding. Supplementation of the older rats with WP, however, was found to restore the neutrophil population during the inflammatory phase of wound healing at the second day after wounding. In both untreated young rats and WP treated older rats, the number of neutrophils was found gradually to reduce to reach a baseline at the eighth day after wounding (Figure 2C,D).

**Figure 3:**
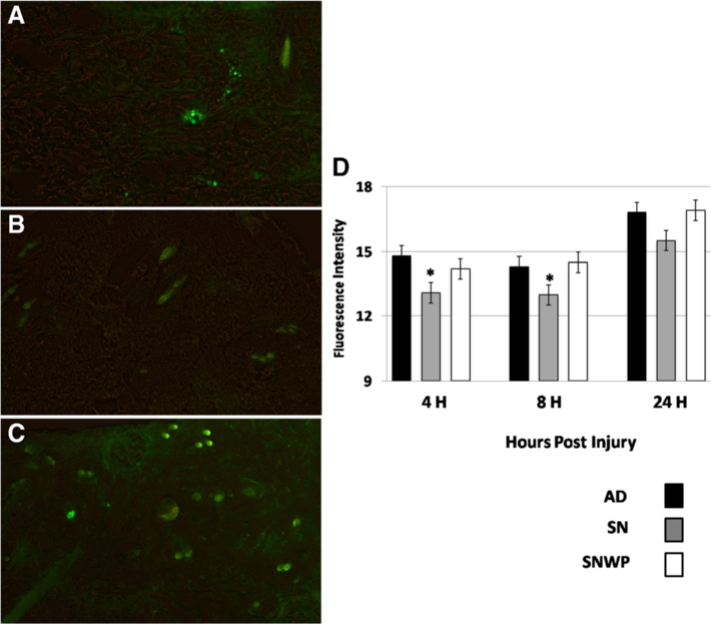
**Phagocytic activity in the inflammatory phase (A: AD, B: SN ,C: SNWP) at 4 hours after wounding. D**: The histogram shows the relative fluorescent intensity of phagocytic activity at 4, 8 and 24 hours after wounding. Values shown are means ± SD.* shows the significance in comparison with the control group.

Fluorescent nanoparticles were applied to evaluate the phagocytic capability of macrophages and neutrophil in the wounded tissues. The results demonstrated that in the older rats phagocytic activity at the wound site was significantly reduced during the early and the middle inflammatory phase periods. In contrast, WP was found to restore the phagocytic activity in the older rats to a normal level throughout the inflammatory phase (4–24 hours after wounding) (Figure 3A-D) and this was obviously reflected in the recovery of the wound healing rate.

### Effect of WP on the proliferation activity of the epidermal cells

**Figure 4:**
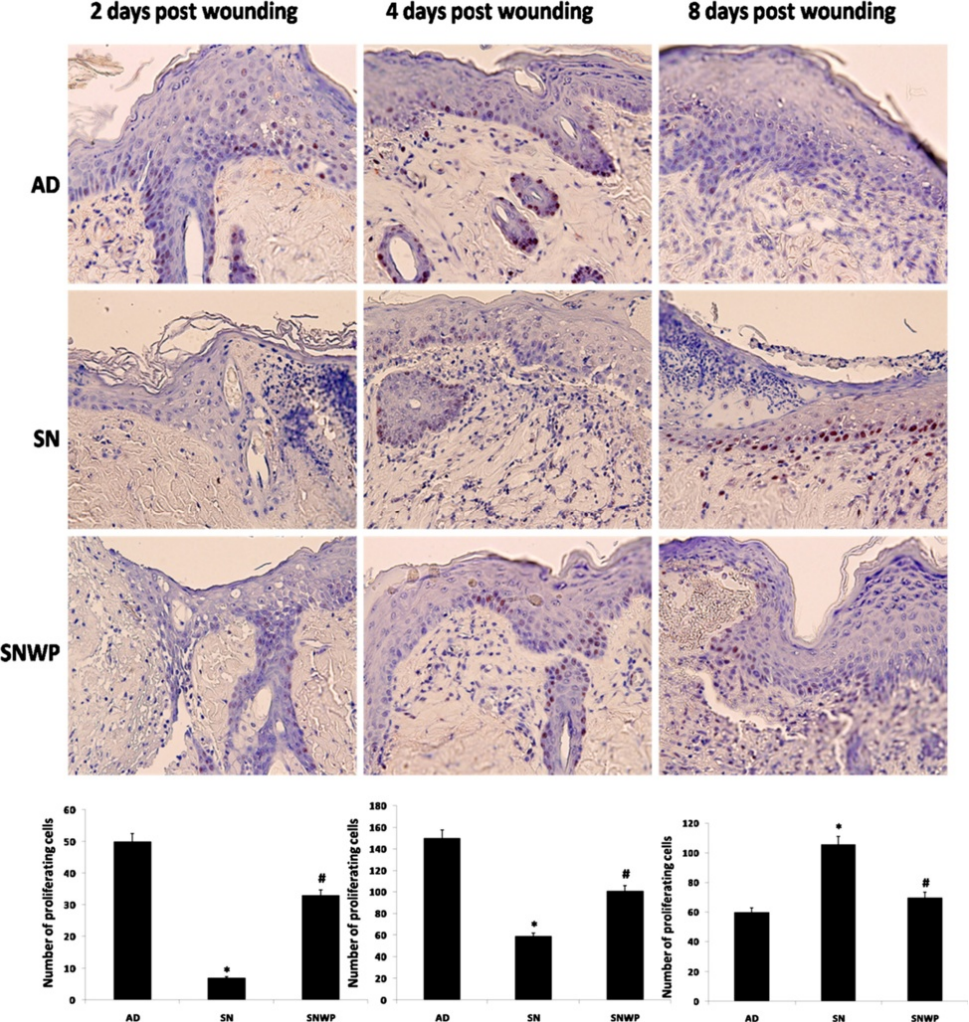
**Skin sections (x 400) stained with anti-PCNA antibodies to show the proliferation activity of the epidermal cells (the proliferating cells are stained with a red colour).** Histograms show the number of proliferating cells at the second, fourth and eighth days after wounding for the different groups. Values shown are means ± SD. *shows the significance in comparison with the control group. # shows the significance in comparison with the older group.

We next followed the proliferation activity of the epidermal cells, representing the second stage of the normal wound healing process. In the young rats this measure was found to increase to reach a peak on the fourth day after wounding before then, as would be expected, decreasing up to the eighth day. In the group of older animals, however, the proliferation activity of the epidermal cells increased right through the observation period. As shown in Figure 4, when the older rats were supplemented with WP proliferation activity was found to behave in a similar way as with the young rat group, peaking on the fourth day after wounding and decreasing thereafter.

### Effect of WP on the re-epithelialization process

**Figure 5:**
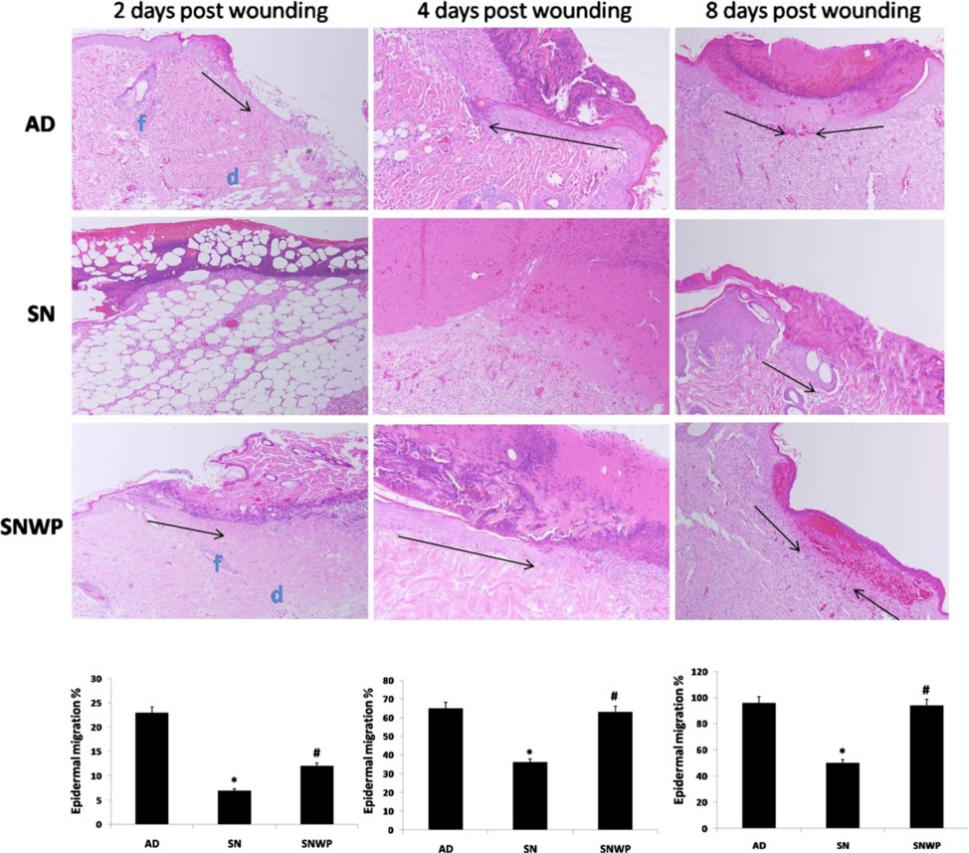
**Representative skin sections stained with H&E to show the migration of proliferating epidermal cells at the second (x 100), fourth (x 200), and eighth (x 100) days after wounding for the different groups.** The epidermal tongues directed toward the entire wound region are shown in these sections. Histograms show the percentage of the original wound size covered by migrating epidermal cells. Values shown are means ± SD. *shows the significance in comparison with the control group. # shows the significance in comparison with the older group.

Histological examination demonstrated that wounded tissues from the older rats appeared disturbed at two days after wounding (Figure 5), while those of the WP-aged rats seemed similar to the younger tissues. Four days after wounding, wound areas in the older rats exhibited an increased extent of wound margin neoepithelia, without obvious epidermal tongues. In contrast, the wound margin epithelia of older rats supplemented with WP, showed an increase in both size and migration (Figure 5) with two epidermal tongues, directed inwards, clearly visible on both sides of the wound. The migration of the epithelial cells at the edge of the wound in WP-aged rats therefore showed a moderate degree of wound closure on the fourth day after wounding, by the eighth day, however,, the wounds of WP-aged rats were completely re-epithelialized, whereas epithelialization was still incomplete in untreated older rats.

### Effect of WP on collagen deposition in the dermis of the wounded tissues

**Figure 6:**
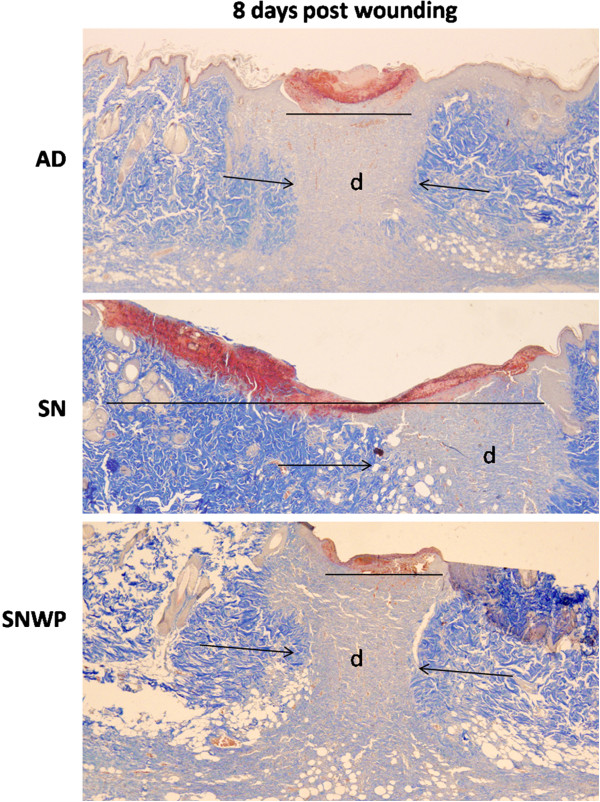
**Mallory Trichrome stain of the skin sections showing collagen deposition at the eighth day after wounding (x 100).** Collagen fibrils appeared symmetrically proliferated and invaded the dermis of the wound region of the young (AD) rat skin (arrows show the direction of the movement of collagen fibres; line shows the narrow wound region eight days after wounding). Unlike the younger rats, disturbed collagen deposition was detected in the older rats (SN) with a wide wound region (line). Collagen fibrils in the older rats supplemented with WP (SNWP) were found to be symmetrically distributed and to have invaded the dermis of the wound region.

One of the most indicative elements of dermal recovery, the rate of deposition of collagen fibrils was investigated in different wound healing phases. Figure 6 shows the reproduction and re-organization of the collagen fibrils in different rat groups on day 8. Mallory Trichrome stain demonstrated that in the young rats there were a moderate number of collagen fibrils and the collagen bundles were organized in a more regular fashion than in the older rats, which tended to exhibit asymmetrically distributed collagen bundles (Figure 6). Dermal regeneration in rats which had been supplemented with WP, however, was characterized by fibroblasts and well-developed symmetrically distributed collagen bundles which were oriented parallel to the epidermal layer from both sides of the wounded regions.

## Discussion

This data confirms the critical role of neutrophil recruitment in the early inflammatory phase of wound healing in older rats. WP was found to recover the wound closure rate in the older rats to a similar level to that of the young rats. These observations are in accordance with those of a previous study that suggested that the Kombucha fungus accelerates the healing quality [25].

Wound healing entails three distinct phases: an initial inflammatory phase, a proliferation phase and, finally, the production and reorganization of the extracellular matrix, leading to tissue repair or regeneration [26-28]. Defects in the inflammatory phase of healing directly cause a failure in the subsequent processes of fibroblast growth and collagen synthesis [29,30]. The inflammatory phase recruits leukocytes that produce growth factors and remove debris from the wound [28]. Impairment of leukocyte recruitment is associated with delayed wound healing [31-33]. Neutrophils release highly active antimicrobial substances, proteinases [34] and inflammatory cytokines which also have crucial roles in the healing of wounds. The precise role of neutrophils in wound healing is still a rich subject of scientific controversy since little is known about exactly how neutrophils affect wound healing. Here, we have investigated neutrophil recruitment during wound healing using an *in vivo* rat model, together with the effects of dietary WP supplementation.

Recent work has suggested that depletion of neutrophils accelerates the healing process [35]. In contrast, in this study, we found that wounds in young rats, which showed normal healing, were infiltrated by greater numbers of neutrophils, especially in the inflammatory stage, followed by a gradual depletion in the next stages. On the other hand, our results point to a marked deficiency in the number of neutrophils in the wounds of aged rats, with this being accompanied by impairment of the healing process. This result is in accordance with Nishio et al., [11] who also argued that the depletion in neutrophils served to impair the rate of healing of wounds in older mice. In addition, Brubaker et al., [7] have argued that reduced neutrophil chemotaxis and infiltration contributes to delayed resolution of cutaneous wound infection with advanced age.

The inflammatory phase of healing can be divided into an early phase, with neutrophil recruitment, and a late phase with the appearance and transformation of monocytes [36]. Normally, neutrophils are recruited to the site of the skin injury and are present for 2–5 days unless the wound gets infected [36]. Some studies, however, have shown that, in some cases, such as with wounds in diabetics, a delayed contribution of neutrophils at the wound site subsequent to the inflammatory stage suppresses the healing process due to the accompanying high inflammation and oxidative stress [35,37,38]. Here we found that neutrophil infiltration of the older dermis delayed to the eighth day post wounding. At this late stage, it is more likely that neutrophils may produce persistently high levels of free radicals and inflammatory cytokines, as suggested by Fulop et al. [39] who mentioned that aging causes multiple defects in PMN function, notably increased production of ROS with high concentrations of inflammatory cytokines induced by a high level of free radicals via NFkB. This may explain the prolonged process of wound closure in rats from the older group: in other words, prolonged wounds remain in a chronic inflammatory state [29] which leads to abnormal wound repair [36]. On the other hand, WP-lactoferrin has an ability to stimulate immune responses involving neutrophils and macrophage cytotoxicity [40]. This further explains the early infiltration of neutrophils in old rats supplemented with WP. Moreover, it is concluded that lactoferrin acts as an anti-inflammatory by regulating the levels of TNF-α and IL-6 [41]. This explains the significant decrease in TNF-α in the serum of old rats supplemented with WP. In addition, increasing the level of the powerful antioxidant, glutathione, by WP supplementation of the older rats (Figure 1D), may have induced oxidative stability, scavenging the free radicals after the inflammatory phase of wound healing. This should suppress the induction of inflammatory cytokines and may encourage the next stages of normal healing.

The alteration of neutrophil functions observed here might be caused by alterations in the signal transduction pathways [39]. Changes in membrane fluidity affect PMN functions, such as chemotaxis and superoxide anion production [42]. Furthermore, neutrophils are necessary for producing MMPs, which further degrade the damaged tissue and produce chemokines, which in turn attract additional neutrophils [11]. Here, WP was found to significantly up-regulate the MIP-1α and CINC-1 mRNA expression in old rats supplemented with WP at 2 days after wounding with respect to old rats, in which MIP-1α and CINC-1 mRNA expression was delayed to the 8^th^ day after wounding. This data is a further evidence on the early and the delayed infiltration of neutrophils in old rats supplemented with WP and old rats, respectively.

Neutrophils are uniquely sensitive to a vast array of chemoattractants including a multitude of chemokines (CINC-1, MIP-1a, MIP-1b, MIP-2, KC, and others). Chemoattractants signals choreograph the transit of leukocytes out of the mainstream of blood and into tissues at sites of inflammation.

The re-epithelialization process is underpinned by keratinocytes at the wound edges and by epithelial stem cells from hair follicles or sweat glands [43,44]. In the present study, wound margin epithelia of older rats supplemented with WP were increased in both their size and the extent of their migration at the edge of the wound, resulting in a moderate closing of the wound by the fourth day after wounding. The release of EGF, TGF-a, and FGF is thought to stimulate epithelial cell migration and proliferation, with keratinocytes then migrating over the provisional extracellular matrix. Once wound closure is achieved, keratinocytes undergo stratification and differentiation to restore the barrier [45]. Our results demonstrated that WP may encourage these successive events of re-epethialization, resulting in a complete covering of the wound region by the eighth day after wounding.

At the beginning of re-epithelialization from the wound edges, neovascularization is activated [46-48]. Neutrophils release mediators such as TNF-α, IL-1ß and IL-6, which serve to stimulate VEGF and IL-8 for an adequate repair response [36]. Binding of VEGF to their receptors on the endothelial cells of existing vessels activates the intracellular signalling cascades [36,49] and cause the endothelial cells to proliferate and migrate into the wound, a process known as 'sprouting’ [50]. In our study, the number of blood vessels and the extent of collagen deposition in the wounded region were significantly reduced in the older rats in comparison to the younger ones, but WP was found to significantly restore these indicators in the older group. Since WP also served to restore the number of neutrophils a positive correlation between the number of neutrophils and the formation of new vessels is proved.

Taken together, our results confirm that neutrophils are more likely to play a central role in the early inflammatory phase of wound healing in the elderly. Depletion of neutrophils in this stage may affect: 1) the secretion of inflammatory cytokines; 2) the production of chemokines; 3) the proliferation and migration of keratinocytes; 4) the initiation of angiogenesis, and 5) the formation and deposition of collagen fibres. Hence, this study has shown that WP may exert a beneficial effect on wound healing in older rats by restoring normal levels of neutrophil infiltration. This data may provide critical insights into future nutritional intervention strategies designed to enhance wound healing in the elderly.

## Abbreviations

CINC-1: Cytokine-induced neutrophil chemoattractant-1; IL-1b: Interleukin-1b; IL-6: Interleukin-6; IL-10: Interleukin-10; GP: Glutathione; MDA: Malondialdehyde; ROS: Reactive oxygen species; SOD: Superoxide dismutase; TNF-α: Tumor necrosis factor alpha; MMP: Matrix metalloproteinases; PMN: Polymorphonuclear cells.

## Competing interests

The author declares that there to be no competing interests.
